# Calprotectin could be a potential biomarker for acute appendicitis

**DOI:** 10.1186/s12967-016-0863-3

**Published:** 2016-04-27

**Authors:** Peter C. Ambe, Daniel Gödde, Lars Bönicke, Marios Papadakis, Stephan Störkel, Hubert Zirngibl

**Affiliations:** Department of Surgery, HELIOS Universitätsklinikum Wuppertal, Witten-Herdecke University, Heusnerstr. 40, 42283 Wuppertal, Germany; Institute of Pathology and Molecular Pathology, HELIOS Universitätsklinikum Wuppertal, Witten-Herdecke University, Heusnerstr. 40, 42283 Wuppertal, Germany

**Keywords:** Acute appendicitis, Negative appendectomy, Calprotectin, Biomarker, Immunohistochemistry

## Abstract

**Background:**

Acute appendicitis is a common cause for a visit to the emergency department and appendectomy represents the most common emergency procedure in surgery. The rate of negative appendectomy however has remained high despite modern diagnostic apparatus. Therefore, there is need for a better preoperative screening of patients with suspected appendicitis. Calprotectin represents a predominant protein in the cytosol of neutrophil granulocytes and has been extensively investigated with regard to bowel pathologies. This study investigates the expression of calprotectin in the lumen of the vermiform appendix of patients undergoing appendectomy for suspected appendicitis.

**Methods:**

Appendix specimens from patients undergoing emergency appendectomy for suspected acute appendicitis were examined. Acute appendicitis was confirmed on histopathology. The qualitative expression of calprotectin in the vermiform appendix specimens was analyzed using specific calprotectin antibodies.

**Results:**

Vermiform appendix specimens from 52 patients (22 female and 30 male) including 11 with uncomplicated and 41 with complicated appendicitis were analyzed. Strong immunostainings were achieved with calprotectin antibody in the lumen of all specimens irrespective of the extent of appendicitis. Immunostaining was negative in the uninflamed appendix.

**Conclusions:**

High calprotectin activity could be demonstrated within the lumen of vermiform appendix specimens following appendectomy for acute appendicitis. The high luminal accumulation of calprotectin-carrying cells could be interpreted as an invitation to study the expression of calprotectin in stool as a new diagnostic aid in patients with suspected appendicitis.

## Background

Abdominal pain is a frequent cause for a visit to the emergency department. Pain to the lower right quadrant may be a sign of acute appendicitis (AA) [1]. However, similar symptoms may be associated with a variety of pathologies [2, 3]. The clinical differentiation of AA from other pathologies of the right lower abdomen can be challenging. Although the clinical presentation and diagnosis of acute appendicitis appear fairly straight forward, the rate of false appendectomy, especially in female patients, is as high as 45 % [4–6]. Due to fear of the consequences of a missed diagnosis, the indication for surgery for suspected appendicitis is liberally made [7]. Thus appendectomy represents the most commonly performed emergency procedures in general surgery [8]. The rate of false appendectomy, especially in female patients, is alarming. [5, 6]. Besides, complications secondary to negative appendectomy might be devastating [9, 10]. The rate of negative appendectomy still remains high despite the use of modern imaging modalities and clinical scoring systems [5, 6, 11]. Thus there is need for a better preoperative screening in patients with suspected appendicitis.

Calprotectin is a predominant protein found in the cytosol of neutrophil granulocytes and represents a documented inflammatory biomarker for bowel pathologies [1, 4, 12, 13]. Calprotectin has been used in the diagnostic evaluation of a number of bowel conditions. Extensive experience with calprotectin has been recorded in the diagnosis and follow-up of inflammatory bowel disease (Crohn’s disease and ulcerative colitis) [1, 13, 14]. We postulated that calprotectin could be an inflammatory biomarker for AA. This pilot study was designed to investigate the qualitative distribution, occurrence, and expression of calprotectin in the vermiform appendix of patients undergoing laparoscopic appendectomy for suspected acute appendicitis.

## Methods

The study was conducted in accordance with the ethical principles of the Declaration of Helsinki and the principles of Good Clinical Practice [15]. Ethical approval was received from the Ethics Committee of the Witten-Herdecke University. A written consent was obtained from all patients prior to surgery.

This is a prospective single-centre, single-blinded study. Patients undergoing appendectomy for suspected AA at HELIOS Universitätsklinikum Wuppertal, Department of Surgery II of the Witten-Herdecke University were randomly drawn from the prospectively maintained database. The corresponding histopathology slides were retrieved from the Institute of Pathology and Molecular Pathology at Helios Universitätsklinikum Wuppertal, Witten-Herdecke University, Germany. Two independent pathologists analyzed the blinded slides.

A standard three-port laparoscopic appendectomy was performed in all cases. Surgery began with an infraumbilical incision for the placement of the camera port. After capnopneumoperitoneum was instilled, two more ports were inserted; 5 mm in the right lower abdomen and 12 mm in the left lower abdomen. Appendectomy was performed using an endoscopic lineal stapler in all cases. The resected vermiform appendix was removed using an endobag. Drains were placed as needed. Antibiotics were given as needed.

### Histological evaluation

Inflammatory changes of the specimens were appraised on hematoxylin and eosin (HE) stained sections. The extent of inflammation was characterized either as uncomplicated (superficial, phlegmonous AA) or as complicated (ulcerative, gangrenous or suppurative AA). Complicated AA was present in cases with mucosa defects, while the mucosa of specimens with uncomplicated AA was intact. Besides, the presence of periappendicitis was registered and where possible, the underlying etiology was documented.

### Immunohistochemistry

The expression of calprotectin in the appendix specimens was assessed by immunohistochemistry using the DAKO Autostainer plus (DakoCytomation) following the manufacturer’s instructions. 3–5 µm sections of formalin-fixed, paraffin-embedded tissue were dried overnight at 37 °C and deparaffinized. Antigen retrieval was performed using the Target Retrieval Solution (Citrate pH 6.1, 10×, DakoCytomation, cat.no. S1699*)* after which the slides were steamed for 30 min. Endogenous peroxidases were blocked by incubation with Peroxidase-Blocking Solution (DAKO REAL ™Peroxidase-Blocking Solution, cat.no. S2023) for 5 min.

Immunostaining for calprotectin was achieved using calprotectin monoclonal mouse antibodies (Thermo Scientific, Clone MAC 387, cat.no. MA5-12213). The specimens were incubated for 30 min with calprotectin-specific primary antibody (dilution 1:500) followed by subsequent incubations with a visualization reagent based on a dextran technology (EnVision + Dual Link System-HRP, DAKO, cat.no. K4061). The EnVision reagent consists of both secondary rabbit anti-mouse antibody molecules and horseradish peroxidase molecules linked to a common dextran polymer backbone, thus eliminating the need for sequential application of link antibody and peroxidase conjugate. Staining was completed by incubation with a substrate-chromogen (Liquid DAB + Substrate Chromogen System, Dako Cytomation, cat.no. K3468) for 2 × 5 min. Enzymatic conversion of the sub-sequentially added chromogen resulted in the formation of a visible brown reaction product at the antigen site. In addition, the nuclei were counterstained with Mayer’s Hematoxylin for 2 min and sealed with coverslips.

### Evaluation of immunohistochemical staining

Two experienced independent pathologists, who were blinded to the clinicopathological data, examined the expression of calprotectin in the stained sections. Immunohistochemical activity was determined in epithelial and inflammatory cells in consideration of the amount of inflammatory cells within the lumen of the vermiform appendix. Staining intensity was graded as negative, weak or strong. The scores of the two pathologists were compared and discrepancies resolved by re-examination to achieve a consensus score.

## Results

**Fig. 1:**
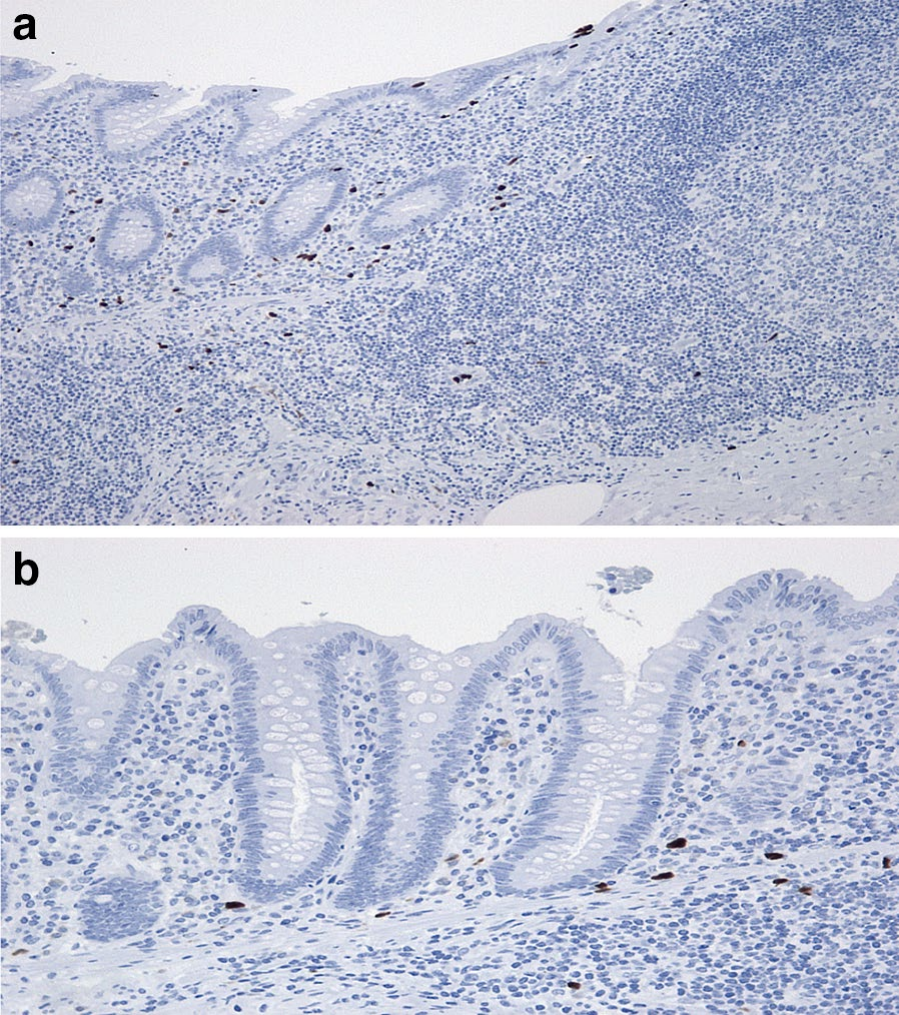
**a** and **b** Immunostaining with Calprotectin antibody showing an uncomplicated appendicitis with unaltered luminal epithelial architecture. **b** detail to **a**. Note the immunohistochemical reaction of neutrophil granulocytes and the absence of immunostaining in the epithelium

Appendix specimens from 52 (22 female and 30 male) randomly drawn patients were analyzed. The mean age of the patients included was 33.6 ± 20.8 years (range 15–77 years). Uncomplicated appendicitis without mucosal defects was diagnosed in 11 cases (21.2 %) including two cases with superficial and nine cases with phlegmonous AA while advanced appendicitis was seen in 41 cases (78.8 %) including 24 ulcerative, seven suppurative and 10 gangrenous AA, Fig. 1a, b.

Mild and severe periappendicitis was recorded in 12 cases (23.1 %) respectively while moderate periappendicitis was seen in 16 cases (30.8 %). AA was associated with abscess formation in five cases (9.6 %). The remaining seven cases (13.5 %) showed no sign of periappendicitis. The underlying etiology of AA was evident in 23 (44.2 %) cases including 20 cases with fecolith, two cases with benign neoplasm of the vermiform appendix and one case of AA secondary to mucocele. The cause of AA could not be found in 29 cases (55.8 %).

**Fig. 2:**
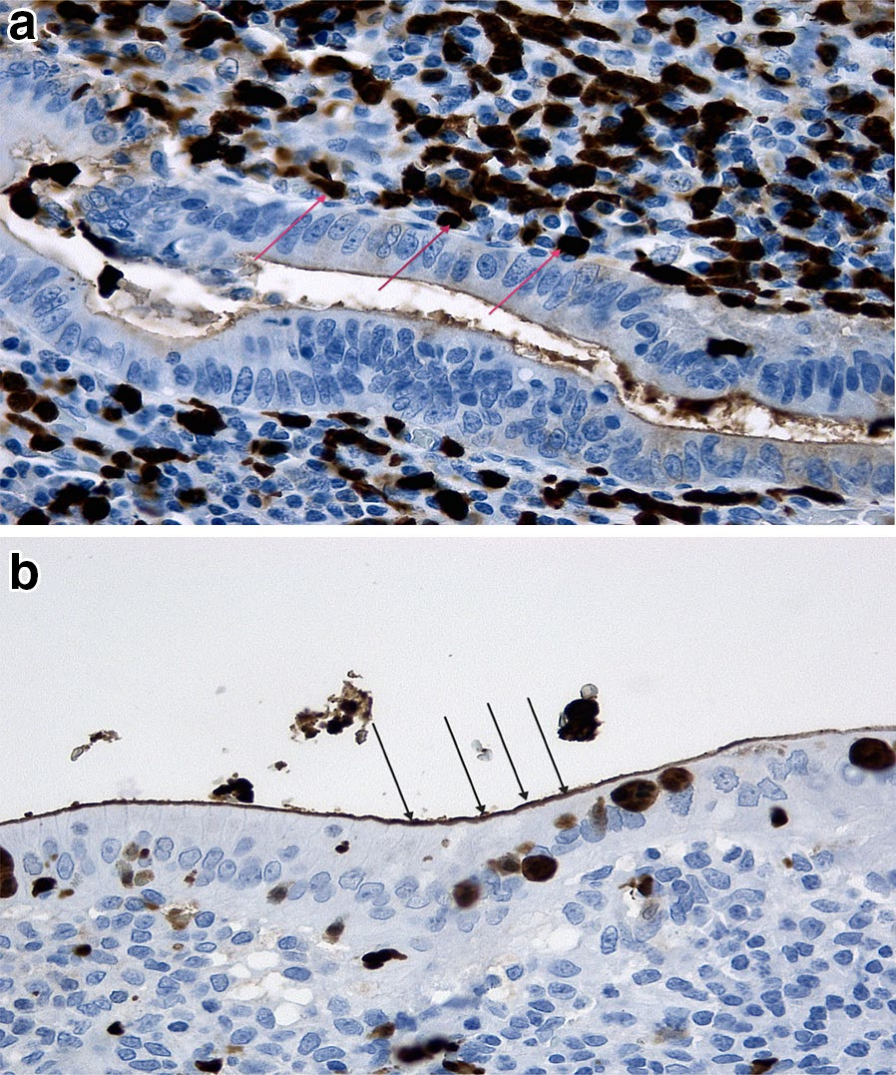
**a** and **b** Immunostaining with calprotectin antibody. Note the strong immunohistochemical reaction (*red arrows* **a**) in the granulocytes and the weak reaction over the apical epithelial membrane (*black arrows*, **b**)

**Fig. 3:**
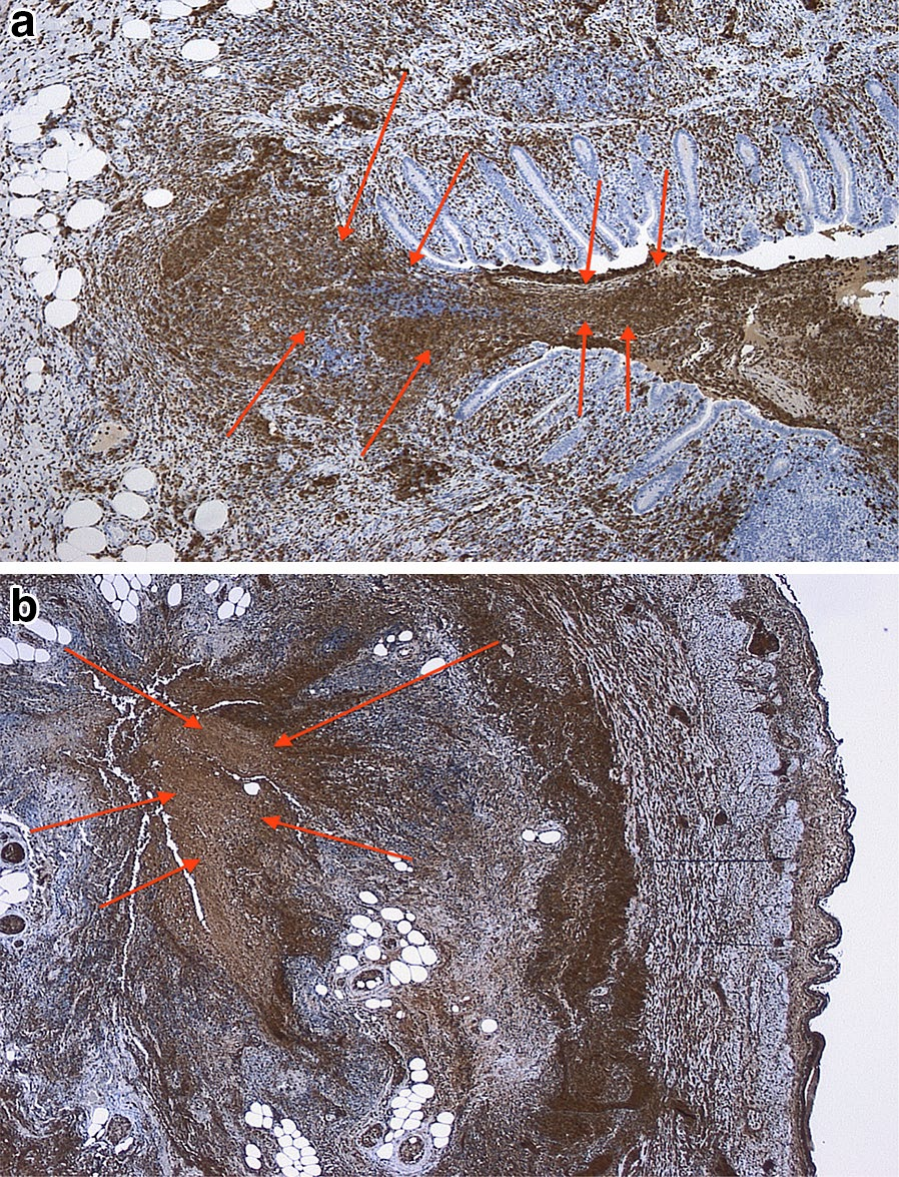
**a** and **b** Immunostaining with calprotectin antibody in a specimen with complicated appendicitis with ulceration and intensive immunohistochemical reaction in the lumen (*red arrows*) of appendix due to massive discharge of inflammatory cells

**Fig. 4:**
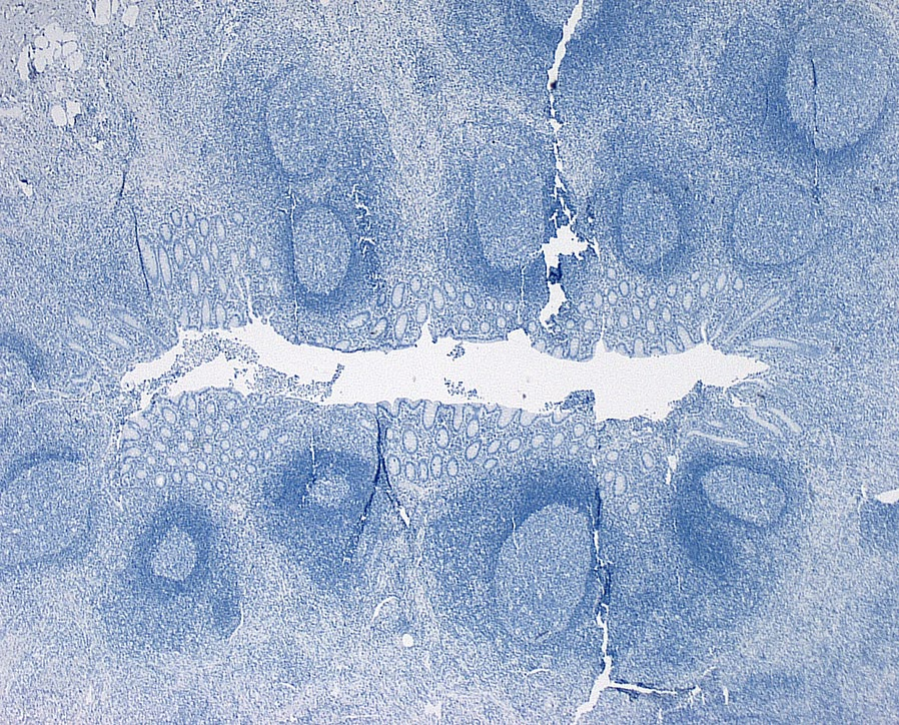
Control using a specimen from an uninflamed vermiform appendix. Note the absence of immunochemical reaction

The intensity of immunostaining of the vermiform appendix with calprotectin antibody was weak in 24 cases (46.2 %), moderate in two cases (3.8 %) and negative in 26 cases (50.0 %), Fig. 2a, b. Excellent immunostaining with calprotectin antibody was achieved in all cases within the appendix lumen, Fig. 3a, b. This finding was independent of the extent of AA. Weak immunohistochemical reaction was observed at the epithelial membrane in all cases. Immunostaining for calprotectin was negative in an uninflamed vermiform appendix specimen (Fig. 4). This control specimen was taken from a patient after right hemicolectomy for colon cancer.

## Discussion

Acute appendicitis is a common illness. However, the clinical differentiation between AA and other potentially less serious conditions might be challenging. Due to fear of the consequences of missed diagnosis, the indication for emergency appendectomy is grossly made. Despite the use of modern diagnostic apparatus, scoring systems, etc. the rates of negative appendectomy remain high [6, 7]. Therefore there is need for a more sensitive preoperative diagnostic aid. This study was designed to investigate calprotectin as a potential biomarker for AA.

Appendix specimens from 52 patients following emergency appendectomy for suspected AA were examined. The qualitative expression of calprotectin in these specimens was investigated via immunohistochemical analysis using calprotectin antibodies. While no or weak immunostaining was recorded within the appendix wall, excellent immunostaining was achieved in all cases both within the lumen of vermiform appendix irrespective of the extent of inflammation.

Calprotectin is a predominant protein found in the cytosol of neutrophil granulocytes and has been extensively investigated with regards to intestinal disorders [4, 14, 16]. The measurement of calprotectin in stool for example is a standard diagnostic work-up in patients with Crohn´s disease, and the levels of fecal calprotectin in these patients have been shown to correlate with disease activity [17, 18]. Therefore, fecal calprotectin is a marker for intestinal inflammation.

The vermiform appendix as an intestinal organ is principally not very different to the small and large bowels. Therefore an inflammation of the appendix as seen in AA could be associated with changes in the expression of calprotectin. This alluring theory was investigated in this pilot study.

*The main findings in this study were the strong immunohistochemical reaction within the lumen of the inflamed appendix, the relatively weak immunostaining over the epithelial membrane and the negative immunostaining in uninflamed vermiform appendix specimens. The finding of strong calprotectin expression in inflammatory cells within the lumen of the vermiform appendix supports the assumption that AA might be associated with changes in fecal calprotectin. It is therefore thinkable, that fecal calprotectin could be helpful in the diagnosis of AA.*

Fecal calprotectin fulfills relevant criteria for a biomarker. It is easy to harvest without the need for an invasive procedure (bowel movement), is robust against enzymatic degradation and stabile at room temperature for more than 7 days [4, 19]. The probably most important feature of fecal calprotectin as a possible diagnostic aid for AA is the possibility of a rapid analysis with results within 15 min using commercially available point of care devices [16, 20, 21].

Taken together, the findings from this pilot study establish a direct association between AA and calprotectin. This study is the first step toward investigating a new biomarker for AA. We would be performing a quantitative analysis of fecal calprotectin in patients with AA and would be able to present our results in the nearest future.

## Conclusion

High calprotectin activity could be demonstrated within the lumen of vermiform appendix specimens following appendectomy for acute appendicitis. The high luminal accumulation of calprotectin-carrying cells could be interpreted as an invitation to study the expression of calprotectin in stool as a new diagnostic aid in patients with suspected appendicitis.

## Abbreviations

AA: acute appendicitis
HE: hematoxylin and eosin
